# Review of Session 7: non-cancer risk

**DOI:** 10.1093/jrr/rrt200

**Published:** 2014-03

**Authors:** Kathryn D. Held

**Affiliations:** Massachusetts General Hospital/Harvard Medical School, Radiation Oncology, Cox 301, 55 Fruit Street, Boston, MA, USA

**Keywords:** heavy ion effects, hematopoietic damage, cardiovascular risks, non-cancer radiation effects, lifespan shortening

## Abstract

Astronauts in space and cancer patients being treated with ion beam radiotherapy can be exposed to charged particle radiations including energetic protons and heavy ions. These charged particles may be more effective than photons in inducing cancer as well as in causing non-cancer effects. The latter include acute damage from large solar particle events to the blood-forming organs and skin, acute and (from heavier ions) late damage to the central nervous system, and late degenerative damage to the lens of the eye and the cardiovascular, circulatory and respiratory systems. The presentations in this session discussed a number of non-cancer effects of protons and heavier charged particles including acute hematopoietic alterations, potentially detrimental cardiovascular and circulatory effects, and lifespan shortening.

## INTRODUCTION

Astronauts on long duration, exploration missions outside low Earth orbit (LEO) are exposed to a unique and complex radiation environment that is quite different from exposures received by terrestrial radiation workers. The space radiation environment includes constant, low-fluence Galactic Cosmic Rays (GCRs)—composed predominantly of highly energetic protons and a small percentage of high-atomic-weight, high-energy (HZE) particles—and sporadic Solar Particle Events (SPEs)—composed mostly of energetic protons. Historically, it has been thought that fatal cancer is the major radiation risk astronauts face from missions beyond LEO [1], and there have been extensive efforts made to develop radiation risk models, such as the NASA Space Cancer Risk model, to estimate cancer risks and associated uncertainties for space missions [2]. However, recent epidemiology studies suggest that degenerative tissue effects, including to the lens of the eye and the cardiovascular and circulatory systems, could also be a concern for astronauts [3, 4]. Risks of both early and late damage to the central nervous system are also a concern [5, 6]; these risks are addressed in a separate session of this meeting. Furthermore, the potential problem of an unpredictably large SPE causing acute radiation syndrome (ARS) that could threaten mission performance continues to exist [5, 7]. This session included presentations discussing acute hematopoietic alterations, potential detrimental cardiovascular and circulatory effects, and lifespan shortening following charged particle radiation exposures.

## DISCUSSION OF PRESENTATIONS IN THE SESSION

The first presentation in the session was given by Dr Olga Smirnova (of the Federal State Unitary Enterprise, Moscow, Russia) on the dynamics of the cellular components of the hematopoietic system following radiation exposure. One of the most radiosensitive tissues/organ systems in mammals is the hematopoietic system. The risk of hematopoietic damage, as part of the ARS, from a large SPE during a space mission has long been recognized [5]. Without sufficient shielding, a large SPE could result in a whole-body dose of over 0.5 Gy over several hours [7]. Although the effects of X-rays and gamma-rays in causing ARS are reasonably well understood, less is known about the hematopoietic effects from an SPE. Also, complicating understanding of acute effects from an SPE is that the protons that are the predominant component of an SPE have a wide range of energies and dose rates within an astronaut's body. Furthermore, with increasing depth in tissue, a fraction, albeit small, of the SPE protons become higher-LET because of the slowing down of higher energy protons. NASA currently sets short-term dose limits for astronaut exposure to radiation, e.g. a 250 mGy-Eq 30-day limit to the blood-forming organ (BFO) [7], but additional understanding of the magnitude and time-courses of changes in various components of the hematopoietic system following radiation exposures could help with the development of effective medical countermeasures and shielding approaches for astronaut protection from an SPE. Dr Smirnova presented her development of biologically motivated mathematical models that describe the dynamics of three major hematopoietic lineages—thrombocytopoietic, granulocytopoietic and erythrocytopoietic—in irradiated humans. The models consider the stages of development of the three lineages and the dose responses of the changes with time in each lineage following irradiation. Dr Smirnova shows that the models agree well with the available human data from photon irradiation, supporting their future application to radiation risk assessment for astronauts.

Four presentations in the session discussed important topics related to the effects of heavy ions on the heart or the vasculature/circulatory system. A substantial volume of epidemiology data has indicated a relationship between high doses of photons, the common terrestrial radiation, and cardiovascular disease or degenerative heart changes. At high doses (>5 Gy), dose-dependent increases in stroke and cardiovascular diseases including pericarditis, coronary artery disease, cardiomyopathy and valvular disease have been shown in patients treated for cancers such as breast cancer and Hodgkin's lymphoma, as reviewed, e.g., in [8 ]. More recently, accumulating epidemiology data from Japanese atomic bomb survivors and other groups, including occupationally exposed individuals, provide evidence of excess risk of circulatory and heart diseases at much lower doses of low-LET radiation and after much longer time intervals, as reviewed, e.g., in [9]. Based on these low-LET epidemiology data, the risk of cardiac and circulatory diseases can now be included in risk models for radiation exposure in astronauts, and that inclusion is now estimated to increase the percentage REID (Risk of Exposure-Induced Death) by about 40% compared with that from carcinogenesis alone [10 ]. However, no information is available on the effects of high-LET, space-like radiations on the heart and circulatory systems in humans, and very little information is available in experimental model systems. Therefore, there is great uncertainty in the relative biological effectiveness (RBE) values for cardiovascular/circulatory diseases used in risk modeling, and improved information on HZE effects at space-relevant doses is critically needed.

Dr David Goukassian of Tufts University School of Medicine, Boston, USA, gave the first cardiovascular-related presentation of the session. The work by his team demonstrates a complex interplay between radiation quality and time after radiation in terms of effects on heart damage-related endpoints and response to a subsequent experimentally induced adverse cardiovascular event, an acute myocardial infarct, AMI. In studies using C57BL6N mice at 9 months of age at the time of total body irradiation, the team showed that after 50 cGy of 1-GeV protons, there were no discernable effects on the heart for up to 3 months, but at 10 months there was a considerable decrease in the relaxation function of the heart. In contrast, only 15 cGy of high-LET, 1-GeV/n iron ions caused several negative impacts on homeostasis and cardiac function at the measured times of 1, 3 and 10 months after irradiation. Subjecting mice to an AMI at varying times after irradiation gave interesting results in that hearts in proton-irradiated animals did not show negative effects in post-AMI recovery, and there may even have been some short-term benefit from proton irradiation, based on molecular markers of cell survival and proliferation. On the other hand, iron ion irradiation appeared to cause significant impairment of the recovery of the heart from an adverse cardiac event, an AMI. Further studies on the underlying mechanisms for these effects are expected to be informative.

Dr Johannes Friess (of the University of Applied Sciences Aschaffenburg, Germany) presented studies that he and his colleagues conducted using cultured primary avian cardiomyocytes exposed to several heavy ion species at GSI Darmstadt, Germany. In this interesting experimental system, the team investigates the electrophysiological responses of cells grown on a microelectrode array and can compare the results of those functional studies with cell proliferation and DNA damage assessed immunohistologically. The electrophysiological studies demonstrated large intersample variations among cells, which precluded demonstration of any effects of doses up to 7 Gy of carbon, titanium or nickel ions, although dose-dependent cell cycle delay and formation of DNA double-strand breaks were seen. Electrophysiological approaches are promising for the insight they could provide on heart function following radiation stress, and it is hoped that more sensitive methods can be developed.

The third cardiovascular talk was given by Dr Dennis Kucik, who represented his team from the University of Alabama, Birmingham, USA. It is thought that endothelium-dependent vasodilation predisposes humans to the development of vascular alterations that can lead to atherosclerosis. This team has been investigating the effect of space-like radiations on development of atherosclerosis in an apolipoprotein E-deficient (ApoE^−/-^) mouse model [11], which is prone to development of atherosclerotic lesions. Mice were exposed to iron ions at a dose that had previously been shown to cause atherosclerotic plaques in this model at longer times after irradiation, then aortic rings isolated from the animals at 4–5 weeks were assessed for relaxation. Compared with unirradiated controls, aortic relaxation was significantly impaired after iron ion irradiation, suggesting that alteration of normal vascular reactivity may precede development of atherosclerotic plaques. Given that atherosclerosis is a major factor in cardiovascular disease, studies such as these that provide insight into the mechanisms of possible effects of radiation could make an important contribution to developing understanding of the risk of cardiovascular diseases in astronauts.

Dr Peter Grabham of Columbia University, New York, USA, presented the last talk on cardiovascular effects of the session, the emphasis of his team being on radiation effects on vasculature. The extensive network of blood vessels, including microcapillaries, in the human body presents a potentially important target for the effects of radiation [12], and it has long been recognized that vascular endothelial cells are targets for radiation-induced damage. Grabham and his colleagues have developed a novel 3D cell culture model using primary human brain microvascular endothelial cells or human umbilical vein endothelial cells grown in a collagen gel, so that (with time in culture) the endothelial cells form capillary-like tubes with central lumens. Depending on the stage of development at which the tube-forming cultures are irradiated, the effects of radiation on growing or mature vessels can be evaluated. The group has shown that both protons and iron ions are equally highly effective at inhibiting vessel formation in immature vessels, but that iron ions are at least four times more effective than protons at disrupting mature vessels. They have extended that work to provide interesting new information on molecular mechanisms underlying the differences. Protons and other ions with LET values ≤ 1 keV/μm inhibit capillary formation by altering the ability of Protein kinase C (PKC)-dependent motile tips to migrate through the collagen matrix to meet other migrating cell processes. On the other hand, ions with LET values > 8 keV/μm affect the later stages of vasculogenesis by causing vessels to fail to complete angiogenesis and form tubular structures. The team has recently published some of these novel data [13].

The last talk presented new data on lifespan shortening following X-ray or carbon ion exposure in mice of varying ages. Previous investigations using female mice have indicated that infants are more sensitive to radiation-induced lifespan shortening than mice irradiated *in utero* or as adults. With the increase in children being treated with charged particle therapy, concern exists as to whether the charged particle exposure could have increased effectiveness compared with photons for the induction of shortened lifespans, which may or may not be due to increased cancer incidence. In the work presented, Dr Shizuko Kakinuma and his colleagues irradiated male and female mice of varying ages with gamma rays or 13–14-keV/μm carbon ions to simulate the entrance LET prior to the spread-out Bragg peak (SOPB) used in ion beam therapy. Their data showed that generally female mice were more susceptible than males to induction of lifespan shortening, and the most sensitive time appeared to be when mice were 1 week old, with less sensitivity *in utero* and as adults, and the RBE for the effect following carbon ion irradiation was ∼ 2. It will be informative to see, in future studies, the pathological analyses to assess whether the effect is mainly due to increased cancer incidence and, if so, whether the spectrum of cancers is the same at varying ages of irradiation. Also of interest will be whether the spectrum of cancers is the same with carbon ions as with gamma rays, or differs following irradiation with the lower LET particle type, as has been shown with higher LET ions [14].

## FUNDING

Preparation of this review was supported by the National Aeronautics and Space Administration (grant number NNX12AB61G) and the Federal Share of Program Income earned by Massachusetts General Hospital on Proton Therapy Research and Treatment Center from the National Institutes of Health (grant number C06 CA059267).
